# Natural Translating Locomotion Modulates Cortical Activity at Action Observation

**DOI:** 10.3389/fnsys.2017.00083

**Published:** 2017-11-07

**Authors:** Thierry Pozzo, Alberto Inuggi, Alejo Keuroghlanian, Stefano Panzeri, Ghislain Saunier, Claudio Campus

**Affiliations:** ^1^Centro di Neurofisiologia Traslazionale, Istituto Italiano di Tecnologia, Ferrara, Italy; ^2^INSERM-U1093, CAPS, Campus Universitaire, Dijon, France; ^3^Unit of Robotics, Brain and Cognitive Sciences, Istituto Italiano di Tecnologia, Genova, Italy; ^4^Laboratory of Neural Computation, Center for Neuroscience and Cognitive Systems, University of Trento, Istituto Italiano di Tecnologia, Rovereto, Italy; ^5^Laboratorio de Cognição Motora, Departamento de Anatomia, Universidade Federal do Pará, Belém, Brasil; ^6^U-VIP Unit for Visually Impaired People, Istituto Italiano di Tecnologia, Genova, Italy

**Keywords:** locomotion, action perception, motor resonance, EEG, translation, body shape

## Abstract

The present study verified if the translational component of locomotion modulated cortical activity recorded at action observation. Previous studies focusing on visual processing of biological motion mainly presented point light walker that were fixed on a spot, thus removing the net translation toward a goal that yet remains a critical feature of locomotor behavior. We hypothesized that if biological motion recognition relies on the transformation of seeing in doing and its expected sensory consequences, a significant effect of translation compared to centered displays on sensorimotor cortical activity is expected. To this aim, we explored whether EEG activity in the theta (4–8 Hz), alpha (8–12 Hz), beta 1 (14–20 Hz) and beta 2 (20–32 Hz) frequency bands exhibited selectivity as participants viewed four types of stimuli: a centered walker, a centered scrambled, a translating walker and a translating scrambled. We found higher theta synchronizations for observed stimulus with familiar shape. Higher power decreases in the beta 1 and beta 2 bands, indicating a stronger motor resonance was elicited by translating compared to centered stimuli. Finally, beta bands modulation in Superior Parietal areas showed that the translational component of locomotion induced greater motor resonance than human shape. Using a Multinomial Logistic Regression classifier we found that Dorsal-Parietal and Inferior-Frontal regions of interest (ROIs), constituting the core of action-observation system, were the only areas capable to discriminate all the four conditions, as reflected by beta activities. Our findings suggest that the embodiment elicited by an observed scenario is strongly mediated by horizontal body displacement.

## Introduction

Human locomotion is possible thanks to central pattern generators allowing a reciprocal activation of flexors and extensors muscle (Grillner and Wallen, 1985). However, cyclical locomotor skill became a decisive step in species evolution when displacement started to be oriented and goal directed. Beside limbs oscillation, backward or forward displacements to avoid a predator or to reach a prey also include a variety of related cognitive processes. Instance of this is spatial navigation toward a goal and the ability to integrate body translation over time that originates from visual flow and vestibular input. Thus locomotion, as a *teleokinetic* behavior (Hess, 1943) is much more than central pattern generator activation producing limb oscillation as when walking on the spot. Real locomotion can thus be described as a goal-oriented action displacing the whole body from one initial position toward a distant spatial goal. In fact, at the perceptual level, a walker on the spot corresponds to an erratic walker without goal, as someone can do for fun in the reverse direction of an airport treadmill, that is a rather atypical visual stimulus.

Despite artificiality of walking without net body translation, forward locomotion seems not a crucial variable in visual processing of biological motion. For example, when a set of point lights (PL) located on the joints of an invisible walker on a treadmill is displayed on a screen the observer reliably distinguishes a human in locomotion in contrast with PL configurations that do not respect normal body metric (Johansson, 1973; Blake and Shiffrar, 2007 for a review). This contrasts with the impossibility to “read” the actions of other species in one’s vicinity when displacement is absent.

Besides this* a priori* considerable visual relevance of body movements for successful interactions with conspecifics and to interpret action of others living beings, a prominent idea regarding motion recognition is related to observer’s motor competencies (Viviani and Stucchi, 1992; Calvo-Merino et al., 2006; Cannon et al., 2014; Quandt and Marshall, 2014; Meirovitch et al., 2015). According to this, the visual input of an observed action would be mapped on to the observers’ own motor repertoire (Gallese et al., 1996; Rizzolatti et al., 2001; Rizzolatti and Craighero, 2004). More specifically, when one observes a living being in motion cortical sensorimotor activity has been proposed to reflect the transformation of perceptual representations to executable actions (Hari et al., 1998; Pavlova and Sokolov, 2003; Pavlova et al., 2003; Oberman et al., 2005; Pineda, 2005; Hirai et al., 2009; Perry and Bentin, 2009). Particularly, it was observed that the visual perception of human action led to an alteration of EEG/MEG activity characterized by a desynchronization of alpha and beta rhythms, which reflects the increase of neural activity within sensorimotor cortices (Pineda, 2005; Pavlidou et al., 2014; Cevallos et al., 2015). Therefore, if horizontal body displacement is a key component of locomotion and if visual perception of kinematic features is tuned by motor representations (Viviani and Stucchi, 1992; Pozzo et al., 2006; Saunier et al., 2008), one may assume different motor cortical activities when displaying real locomotion compared to a walker with no net translation.

Traditional protocols, although interesting for examining biological motion recognition processes, presented a strong limitation to address this question. Indeed, most of studies either manipulated the exposure duration of the animation (Thornton, 1998; Poom and Olsson, 2002), embedded the PL in an array of dynamic noise dots (Cutting et al., 1988; Bertenthal and Pinto, 1994; Ikeda et al., 2005; Hiris, 2007) inverted PL (Sumi, 1984) or compared a translating PL with the translation of an object at constant velocity (Peuskens et al., 2005). Although systematic investigation of the role of such ecologically valid component of action is lacking, several experiments however reported that extrinsic motion makes PL displays more natural and easily recognizable (Johansson, 1973; Proffitt et al., 1984; Pavlova and Sokolov, 2003; Thurman and Lu, 2013).

The current study tests the hypothesis that motor experience related to natural translating walking modulated the sensorimotor alpha and beta rhythms. If motion recognition relies on the transformation of seeing in doing and its expected sensory consequences, a significant effect of translation compared to centered displays on sensorimotor cortical activity is expected. We thus collected EEG from participants during the observation of different locomotor patterns, manipulating the gestalt (a scrambled displays that consists of the same amount of absolute motion but lacks a body structure vs. a coherent global body configuration) and the motor/kinematic (walking on a treadmill with no net translation vs. natural translating locomotion) of the display. More specifically, we aimed at verifying that translated scrambled display (that modifies only the body structure but keeps the biological kinematic) would produce sensorimotor spectral perturbations in EEG signal and that the translation cue on its own can link the visual input with the action system.

## Materials and Methods

### Subjects

Thirteen right-handed volunteers (7 females, 6 males, mean age: 27, standard deviation: 3.5), with normal or corrected to normal vision, took part in this study. All participants provided written informed consent before the experiment began. The experimental protocol was approved by the local ethical committee ASL-3 (“Azienda Sanitaria Locale”, local health unit), Genoa, and was in agreement with the Helsinki Declaration of 1975, as revised in 1983.

### Experimental Protocol

Participants were presented with point-light animations (PLAs) built from motion capture data. We used a VICON Motion Capture System with 10 cameras to record the movements, at 100 Hz sampling frequency, of an actor walking naturally (length of recording: 4.5 s). The actor had 13 passive infrared reflective markers placed at the main joints and other landmarks following the VICON Plugin Gait Full Body Template (Eltoukhy et al., 2012): LBHD (left back of the head, roughly in a horizontal plane of the front head markers), LSHO (acromio-clavicular joint), LELB (left outer elbow, lateral epicondyle approximating elbow joint axis), RELB (right outer elbow), LWRA (left wrist bar thumb side), RWRB (right wrist bar pinkie side), LPSI (over the left posterior superior iliac spine), LKNE (lateral epicondyle of the left knee), RKNE (lateral epicondyle of the right knee), LANK (left outer ankle, lateral malleolus along an imaginary line that passes through the transmalleolar axis), RANK (right outer ankle), LTOE (left toe, over the second metatarsal head, on the mid-foot side of the equinus break between fore-foot and mid-foot), RTOE (right toe). These data were processed through Matlab scripts (Mathworks Inc., Natick, MA, USA) to build the stimuli displayed using functions from the Psychophysics Toolbox (Brainard, 1997) on an LCD monitor, with a refresh rate of 60 Hz. Point-lights were white against a black background.

**Figure 1:**
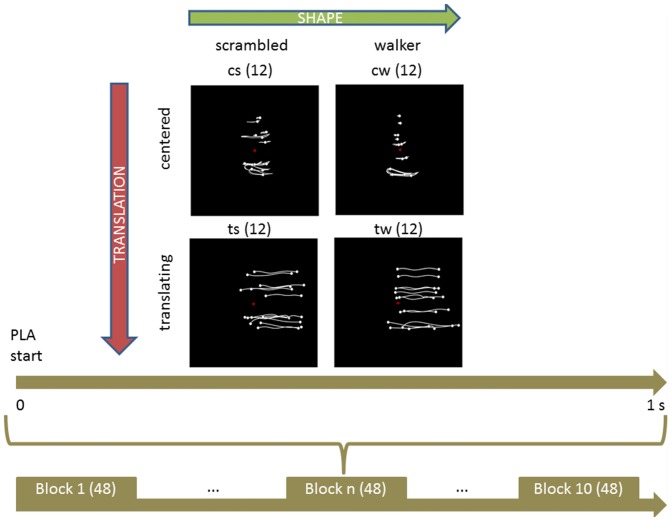
Experimental protocol and stimuli displays. Upper part shows the four categories of stimuli. Body joint trajectories (in white) are depicted on black screen for illustrative purposes but were not visible during the actual experiment. From left to right (upper horizontal green arrow), stimuli shape change from a scrambled structure to a real walker body structure. From top to bottom (left lateral yellow arrow), stimuli keep the same shape but change from a centered to a translated display. *Abbreviations*: cs and cw for centered scrambled and walker respectively; ts and tw for translating scrambled and walker. The trajectories of the markers during the whole point light animation (PLA) are shown. The lower part sums up the experimental protocol. During each of the 10 experimental blocks of 48 trials (in parenthesis), every condition was randomly presented 12 times (upper part, in parenthesis) for 1 s. During each trial, subjects were told to anchor their gaze on the red cross displayed in the middle of the screen.

Four types of stimuli were created: a centered walker, a centered scrambled walker, a translating walker and a translating scrambled walker (see Figure 1), which will be now on referred to as cw, cs, tw and ts, respectively. During the centered conditions the stimulus stayed with its barycenter in the middle of the screen (visual angle approximately between 0 and 9 degrees); during the translating conditions the stimulus moved its barycenter from the middle always to the right of the screen (visual angle approximately between 0 and 18 degrees). Each PLA was 1 s long because of the visual processing duration of such stimuli that occurs within a temporal window of 1000 ms after the display onset (see Hirai et al., 2003; Jokisch et al., 2005; Krakowski et al., 2011; Saunier et al., 2013). In order to avoid a possible bias in the results due to the initial starting positions of the point-lights, each PLA had 10 different starting positions obtained by shifting the animation by steps of six frames (see Hirai et al., 2003). The cw animation was built by translating all the dots of the tw animation by the opposite of the vector defining their center of mass with respect to the center of the screen, at each frame: the cw animation looked like a person walking on a treadmill. The cs animation was built by changing randomly the initial positions of the dots in the cw animation but keeping their velocity vectors unchanged; the dots’ trajectories were constrained to remain inside the vertically-oriented rectangle in which the cw animation was inscribed. This constrain did not affect the velocity vectors of dots in cs and ts conditions. The ts animation was built by changing randomly the initial positions of the tw animation in an analogous way. Therefore, each stimulus was obtained by combining two factors with two levels each: shape (either walker or scrambled) and translation (that could be present or absent).

During the experiment, participants were sitting comfortably in a darkened room, in front of the screen where PLAs were displayed, approximately 60 cm apart. The experiment was organized in 10 blocks. Each block consisted of 48 PLAs (12 of each of the four types) presented in pseudo-random order. In order to avoid possible expectation effects due to extremely regular timing in the presentation of the stimuli, the inter-stimulus interval (ISI) varied randomly in length between 2 and 4 s (uniform distribution). In each block, a random number of animations (between 2–4) changed color from white to green during 250 ms. This change of color occurred in a randomly determined period of the animations. Hereafter, these changing-color stimuli will be referred to as odd stimuli. Once an odd stimulus was presented, after a random number of stimuli a question was presented on the screen asking the participant which was the last animation among the four types that changed color. Since the question arose randomly, it was possible that between two odd stimuli no question was presented. Therefore, the number of questions was variable across blocks, and was less than or equal to the number of odd stimuli presented in the respective block (one in case of two odd stimuli or four in case of four stimuli, in one block). EEG traces corresponding to odd stimuli were discarded from analysis. Participants gave their answers through a keyboard, by pressing a key number between 1, 2, 3 and 4, corresponding to cw, tw, cs and ts, respectively. The four types of stimuli were clearly identifiable, and each participant learned the correspondence between key numbers and stimuli during a training session before starting the recording session. The training consisted first in displaying the stimuli and the associated number: participants were allowed to replay it as many times as they needed to learn the correspondence between numbers and animations. Then, subjects were tested in a short experimental block (12 animations, with three odd stimuli, and two questions) to verify they learnt the correspondence and understood the task. Participants were allowed to replay this second step, or to go back to the first part in case they did not learn the correspondence between PLAs and numbers. Participants did not receive feedback on their performance during the actual experiment, while they did during the training session instead.

### Electrophysiological Data Recording

The electroencephalogram (EEG) was recorded from 62 Ag/AgCl active electrodes (actiCAP, Brain Products, Munchen, Germany) placed on the scalp, mounted on a cap according to the international 10-20 system. The EEG was amplified with two BrainAmp MR plus amplifiers (Brain Products), digitized at 1000 Hz. The recordings were referenced to electrode FCz. Impedances of all electrodes were kept below 15 kOhms.

### Data Pre-Processing

Raw EEG signals were band-pass filtered between 0.16 and 45 Hz through a Butterworth filter as implemented in Brain Vision Analyzer software (Brainproducts). Data were down sampled to 250 Hz and then imported into EEGLAB software (Delorme and Makeig, 2004) for further analyses. A visually inspected artifact removal was performed based on the topographical and spectral distribution and on the time series of the independent component calculated with the ICA algorithm implemented by EEGLab. After artifact cleaning the signal, the percentage of removed events was 7 ± 1% (mean ± SD) for the cs, 6 ± 4% for cw, 6 ± 3% for cs and 7 ± 2% for tw, therefore, for each condition, the number of removed events was on average lower than 5% and with a small variability among all subjects. Data were re-referenced to the common average reference (CAR) and epochs from −400 (as the best compromise to get at the same time a long enough time window and a clean baseline) to 1000 ms with respect to stimulus presentation (time = 0) were then extracted.

### Evaluating ERSP

For each epoch, Fast Fourier Transform (FFT) was applied to partially overlapping time segments: each segment was 256 ms long (64 time points) and each shifting step was 8 ms. A 16 points zero-padding and a Hanning-window tapering were employed, respectively to increase smoothness in ERSP estimation and to limit edge effects. To get a clean estimation of baseline activity, the period between −200 ms and −10 ms was adopted. Event related spectral perturbations (ERSPs) were calculated as event-related power variations (in dB) compared to the specified baseline (Makeig, 1993). ERSPs were then mediated across epochs for each condition, considering times from 200 ms before the stimulus to 850 ms after the stimulus (8 ms resolution) and frequencies from 4 Hz to 32 Hz (approximately 0.5 Hz resolution). Then, based on previous literature, the EEG spectral perturbations were separately evaluated in the theta (4–8 Hz), alpha (8–12 Hz), beta 1 (14–20 Hz) and beta 2 (20–32 Hz) frequency bands. For each subject and condition we used EEGLab to verify that inter trial coherency was significant, therefore spectral modulations were consistent among trials.

### Statistical Analysis

Statistical analyses were performed using the R software (R Core Team, 2017). We considered the average ERSP for three regions of interest (ROIs), namely Ventral (PO7, PO8, P7, P8), Dorsal-Parietal (P1, P2, Pz) and Inferior-Frontal (F7, F8, FT7, FT8, F5, F6). These regions have been selected because they are involved in biological motion perception: Ventral ROI mainly for shape encoding (Grossman and Blake, 2002; Hirai et al., 2003; Krakowski et al., 2011; Saunier et al., 2013), Parietal for decoding and integration of shape and kinematic features (Saygin et al., 2012), while Inferior Frontal reflecting motor resonance mechanism (Cochin et al., 1999; Saygin, 2007).

Spectral modulations of cortical activity are possible neural correlates related to motion perception. Particularly, the theta band is selective to shape discrimination whilst the alpha and beta bands are involved in matching the visual input to motor repertoire (i.e., here referred to “motor resonance”). For instance, the shape of the observed stimulus affects theta frequency band (Urgen et al., 2013). Otherwise, alpha band is affected by both movement perception (Capotosto et al., 2009; Zumer et al., 2014) and execution (Cochin et al., 1999). Finally, the motor characteristics of the observed stimulus specifically affect beta bands (Meirovitch et al., 2015). Indeed, beta 1 is involved in the long-range synchronization of distant areas composing a visuo-motor network, in addition to the matching of perceived stimuli onto the motor repertoire (Engel and Fries, 2010; Kopell et al., 2011). Further, Beta 2 is involved in the process of action selection and monitoring (Botvinick et al., 1999).

#### Comparing ERSP between Conditions

We tested if visual stimuli elicited different neural activities measured through EEG spectral perturbations. For each subject, ROI and frequency band, we extracted the extreme value, i.e., the maximum or the minimum ERSP, for each ROI and band considered: increased mental activity is generally reflected by a power increase (synchronization) in the theta band and by a power decrease (desynchronization) in the alpha and beta bands. Therefore for theta we selected the maximum, while for other bands the minimum (for further details about the procedure of extreme selection, see *Supplementary Statistical Analysis*). To establish an order in intensity and timing of different spectral perturbations, we applied repeated measures analysis of variance (RM-ANOVA), investigating the effects of ROI (Ventral, Dorsal-Parietal and Inferior-Frontal) shape (walker, scrambled), translation (centered, translating), as well as their interaction on the extreme of ERSP and on their latencies. *Post hoc* tests were conducted using paired two-tailed *t*-tests and *P* < 0.05 after Bonferroni correction (to reduce the risk of false positives, we considered all pairwise comparisons between three ROIs and four conditions and thus resulting in a correction factor of 18) were retained as significant. Considering the number of discarded events among subjects in each condition, standard deviations resulted quite low (ranging from 1% to 4% depending on condition), indicating that discarded events were quite uniformly distributed among all subjects. Therefore, in the performed RM-ANOVA *post hoc* we used one averaged value for each subject, frequency band and condition, without any specific weighting procedure.

ANOVAs were used to establish if a difference existed among the magnitudes or the latencies of corresponding to the extreme values. Then, *post hoc* comparisons were used to establish which latencies (amplitudes) were lower/larger, i.e., to establish an order (or hierarchy) between different values. For example, if the extreme value was larger in one scalp area than in another, we inferred that intensity of the activation (estimated as the extreme value) was larger in the first area; if the latency of the extreme value in one area was lower than in another area, we inferred that the timing of the first area was earlier.

#### Predicting Visual Stimuli Using ERSP in Frequency Bands

To draw more reliable functional conclusions, we applied a multinomial logistic regression model (Press and Wilson, 1978) which allows to overcome EEG a-specificity by filtering significant results provided by classical ANOVA followed by *post hoc* comparisons through a greater constraint. We thus searched not only for significant differences between conditions, but for any feature that allows predicting, based on EEG spectral features, the structure (walker or scramble) and kinematic (with or without extrinsic movement) characteristics of the visual input. Moreover, this allows establishing the different weight of body shape (walker or scrambled) and kinematic (centered or in translation) in eliciting motor resonance.

Traditional statistical analyses rely only on raw comparisons between distributions across all trials and subjects of different groups or conditions, merely based on means and standard deviations: describing properties of a sample using these two parameters can however lead to miss information related to subject specificity. To overcome this limitation, we used a Multinomial Logistic Regression model that better takes into account inter subject variability (going beyond group level comparisons and allowing to predict for each single subject the probability of observing a certain kind of stimulus given recorded EEG activity in different frequency bands) and does not assume a normally distributed sample. Moreover, we aimed to use the model as a predictor of the visual stimuli from the single subject measured ERSPs. In this way, we went beyond statistical comparisons between ERSPs and built a classification model that can decode information extracted from EEG giving the probability of an observed stimulus as a continuous function of the measured ERSP. We used multinomial logistic regressions (MLRs), mainly for two reasons: first, they are more generalizable, robust and provide better or comparable performance than linear classification models (Press and Wilson, 1978). Second, they allow not only distinguishing different conditions as classical clustering based approaches, but also allow testing the contribution of each band in decoding visual stimuli. For consistency with ANOVAs and *post hoc* comparisons, the model was fitted considering stimuli as dependent variable and ERSP values of all subjects as predictor, considering one value for each subject, ROI and frequency band.

**Figure 2:**
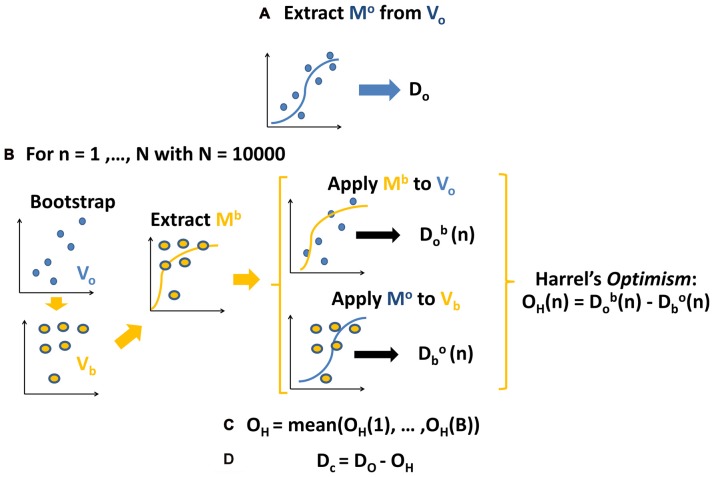
Schema of the applied procedure for fitting and validating the models. See text for further details. **(A)** Calculate the coefficients and initial estimation of Somers’ D for original model fitted to the original ERSP data. **(B)** For 10,000 bootstraps of original data: calculate coefficients of model fitted to bootstrapped data, apply it to original data and original to boostrapped data, computing the difference between respective Somers’ D, i.e., Harrell’s optimism. **(C)** Average Harrell’s optimism over all bootstraps. **(D)** Obtain a corrected estimation of Somers’ D by subtracting Harrell’s optimism from the initial estimation.

The first step (Figure 2A) consisted in extracting the model M^o^ (using a multinomial logistic regression function) from the original values V_0_ (ERSP data). Then we calculated its predictive ability and discriminative performance using Somers’ D index (D_o_) that ranges from 0 to 1 (Somers, 1962). Predictive ability of the model refers here to the inverse process that starts from the ERSP of each subject and comes back to the corresponding visual stimulus. Small D values indicate random predictions unable to discriminate visual conditions; high D values indicate perfect predictions and condition identification.

As shown in Figure 2B, the reproducibility of discriminative performance was verified by a validation based on bootstrap technique (Harrell, 2015), which is based on re-estimation of model parameters on data which were repeatedly randomly sampled with replacement by the original data set and which was found to provide better results than classical cross-validation techniques (Steyerberg et al., 2001). Specifically, we assessed the robustness of the predictive ability of the model with a different set of data V_b_ obtained by bootstrapping original data (V_o_ from which we extracted the model M_b_ (Figure 2B, left part). Then we calculated the D index obtained by crossing the models and the data from which they were extracted: on one side we applied M^o^ to V_b_ obtaining D_b_^o^ (Figure 2B, middle part); on the other side we applied M^b^ to V_o_ obtaining D_o_^b^. At last, as a measure of the reproducibility of model’s performance (Figure 2B, right part), we calculated the difference between the two D indices D_b_^o^ and D_0_^b^, i.e., the Harrel’s *Optimism* O_H_ (Harrell, 2015). We repeated this step 10,000 times, thus getting a distribution of O_H_, i.e., of the differences between D_b_^o^ and D_o_^b^.

We then (Figure 2C) calculated the average of this distribution thus obtaining the *Optimism* O_H_ which is an index describing the bias present in the initial estimation of the model’s performance: the closer is the average *Optimism* to 0, the more reproducible is the performance of the model.

In the last step (Figure 2D) we subtracted O_H_ from the initial estimation of model’s performance D_o_, thus obtaining a corrected and unbiased performance estimation D_c_ (for further details, see *Supplementary Statistical Analysis* and Harrell, 2015). We checked the potential value of ERSP in decoding body shape (cw and tw vs. cs and ts) or translation (tw and ts vs. cw and cs). Moreover, we investigated the respective weight of shape and translation in classifying all visual stimuli at the same time.

To sum up, using multinomial logistic regression model allowed us overcoming the limitations of classical group statistics (Press and Wilson, 1978). On one hand the models selected the most robust effects among those showed by classical ANOVA followed by *post hoc* comparisons, specifically highlighting the peculiar role of cortical areas in the integration of stimuli’ form and motion. On the other hand, the models made it possible to infer from ERSP in different EEG frequency bands the probability of observing a certain kind of stimulus not only at a group, but also at the single subject level. Therefore having stronger (de)synchronizations in specific bands, for each single subject, increased the probability of observing a specific stimulus. At last, the selected class of models allowed us to establish an order between the considered experimental conditions, providing the strength of the “jump” from each condition to another one.

## Results

**Figure 3:**
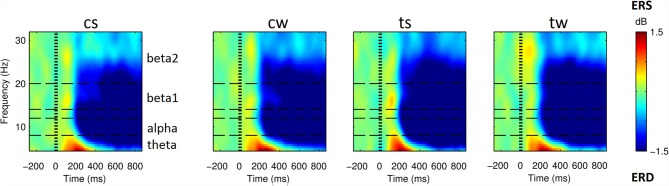
Spectrograms obtained from superior parietal ROI (SP) for the four visual stimuli. From left to right, cs and cw for centered scrambled and walker; ts and tw for translating scrambled and walker). Event related spectral perturbations (ERSP) are expressed at different times (*x*-axis) and frequencies (*y*-axis) as in dB, i.e., Log(Power(t)/Power during baseline). Blue indicates event related desynchronizations (ERD), i.e., power decreases; green indicates null variations, while red indicates event related synchronizations (ERS), i.e., power increases. SP area was selected for representative purposes: in all the ROIs and conditions we found a similar pattern with an initial ERSs, mainly involving low frequencies, followed by an extended ERD mainly involving high frequencies (see also Supplementary Results).

As shown in Figure 3 (see also Supplementary Figure S1), all the visual stimuli produced in all the considered ROIs an early event related synchronization (ERS) and a later Event Related Desynchronization (ERD). ERS spanned approximately from around 100 ms and 400 ms after the stimulus onset and mostly involved the theta band (4–8 Hz). ERD spanned approximately from 200 ms to the end of the considered epoch and mostly involved alpha (8–12 Hz), beta 1 (14–20 Hz) and beta 2 (20–32 Hz) frequency bands.

### General Trend of ERSP for Shape and Translation Effects

**Figure 4:**
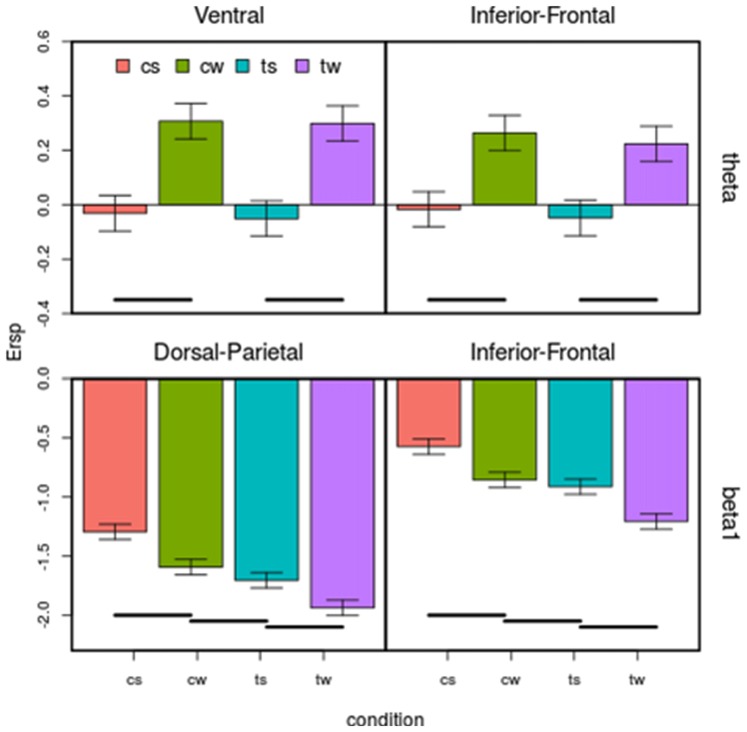
Effect of the four visual stimuli on the four regions of interest (ROIs) considered for both theta and beta bands. Each subplot represents a ROI. On *x*-axis are visual conditions (cs, cw, ts, tw, same abbreviation than in Figure 1); on *y*-axis are ERSP in dB. Bars correspond to means and standard errors. Horizontal lines correspond to significant differences (*p* < 0.05). Top: in Ventral (top left), theta is affected by the main effect of shape, synchronizing more for walker than for scrambled shape in Ventral and in Inferior-Frontal. Bottom: both in Dorsal-Parietal (left) and in Inferior-Frontal (right) beta 1 is affected by shape and translation at the same time, progressively desynchronizing in centered scrambled, centered walker, translating scrambled and translating walker.

Based on ANOVA and *post hoc* comparisons (see Table 1 and Supplementary Figures S2–S4), theta and beta 1 were modulated by shape depending on cortical regions (see green rows in Table 1, Supplementary Figure S2), while alpha and beta bands by translation (see red rows in Table 1, Supplementary Figure S3). For what concerns the shape effect, as shown on top-left of Figure 4, in Ventral ROI, theta showed higher synchronizations for walker (cw and tw) than for scrambled (cs and ts) shape (*t*_(12)_ = 12.33, *P* = 0.0000005): this was found also when separately comparing centered walker with centered scrambled (left, *t*_(12)_ = 8.73, *P* = 0.00002) and translating walker with translating scrambled (right, and *t*_(12)_ = 11.91, *P* = 0.0000006). A similar pattern was found in Inferior-Frontal (top-right Figure 4, *t*_(12)_ = 19.42, *P* = 0.000000002), also when separately comparing cw with cs (left, *t*_(12)_ = 11.28, *P* = 0.000001) and tw with ts (right, *t*_(12)_ = 19.56, *P* = 0.000000002). Conversely, in Dorsal-Parietal (bottom-left Figure 4), walker produced deeper beta 1 desynchronizations than scrambled shape (*t*_(12)_ = 12.33, *P* = 0.0000005), also when separately comparing cw with cs (left, *t*_(12)_ = −20.02, *P* = 0.000000001) and tw with ts (right, *t*_(12)_ = −25.16, *P* = 0.0000000001). A similar result was found in Inferior-Frontal (bottom-right Figure 4, for walker vs. scrambled *t*_(12)_ = 32.22 *P* = 0.000000000006; left for cw vs. *cs*_(12)_ = −21.63, *P* = 0.0000000007; right for tw vs. ts *t*_(12)_ = −19.36, *P* = 0.000000003). For what concerns translation effect, as shown on bottom-left Figure 4, in Dorsal-Parietal beta 1 showed deeper desynchronizations for translating than for centered stimuli (*t*_(12)_ = −34.07, *P* = 0.0000000000008, for ts vs. cs *t*_(12)_ = −34.71, *P* = 0.000000000003 and for tw vs. cw *t*_(12)_ = −22.49, *P* = 0.0000000004). A similar result was found in Inferior-Frontal (for translating vs. centered *t*_(12)_ = −38.07, *P* = 0.0000000000008, for ts vs. cs *t*_(12)_ = −38.33, *P* = 0.0000000000008 and for tw vs. cw *t*_(12)_ = −16.50, *P* = 0.00000002). Translating stimuli produced also deeper alpha desynchronization in Dorsal-Parietal *t*_(12)_ = −5.65, *P* = 0.001 (See Supplementary Figure S3). Interestingly, both Dorsal-Parietal (see bottom-left Figure 4, *t*_(12)_ = −7.32, *P* = 0.0001) and Inferior-Frontal beta 1 (see bottom-right Figure 4, *t*_(12)_ = −6.89, *P* = 0.0002) showed a deeper desynchronization for translating scrambled than for centered walker.

**Table 1:** Results of ANOVAs on event related spectral perturbations (ERSP) extreme values.

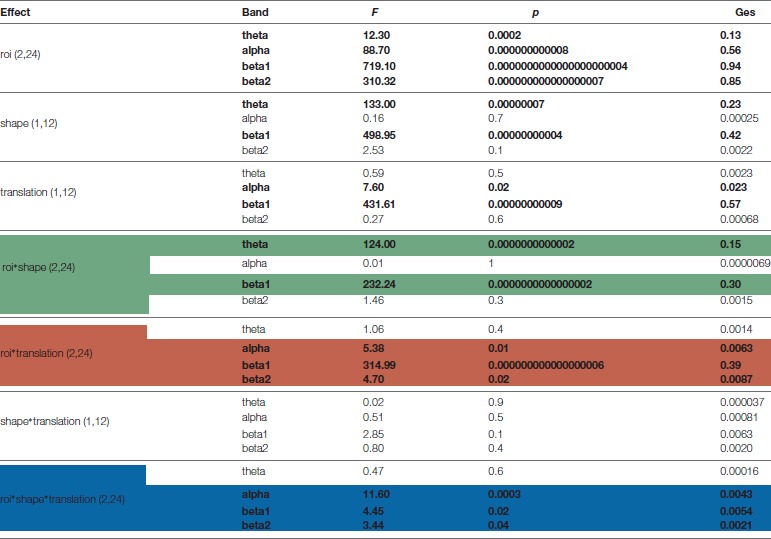

*Columns represent respectively: experimental effect, frequency band, F value p values and generalized eta squared index. Degrees of freedom are in parentheses. Significant effects (*P* < 0.05) are in bold. In green bands are shown which more responding to shape, in red bands are shown which more responding to translation effect, while in blue bands are shown which responding to their interaction*.

### Comparing the Latencies of Extreme ERSP Values

For all bands, the latencies of extreme ERSP values followed, as expected, a caudal-to-rostral order: extreme values occurred in Ventral, then in Dorsal-Parietal and finally in Inferior-Frontal. The average peak latencies for different ROIs spanned from 110 ms to 544 ms for theta, from 119 ms to 551 ms for alpha, from 142 ms to 574 ms for beta 1 and from 154 ms to 586 ms for beta 2 (see also Supplementary Figures S5, S6).

### ERSPs in Specific Frequency Bands Can Predict the Observed Visual Stimuli

**Figure 5:**
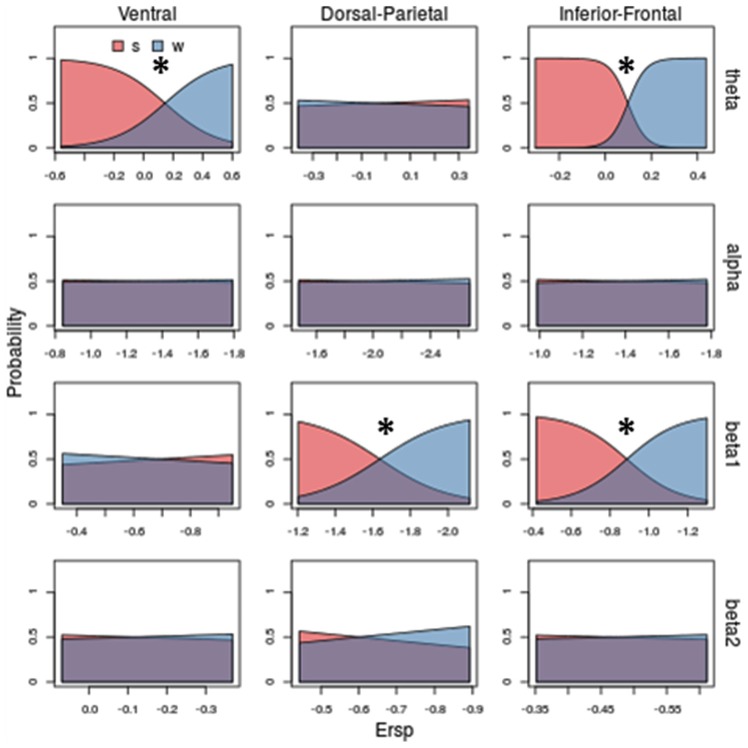
Model prediction on stimulus shape (scamble vs. walker) based on ERSP. Rows and columns of subplots correspond respectively to different frequency bands and ROIs. In each subplot, on *x-*axis are ERSP in dB; on *y*-axis are probabilities of observing a scrambled (s) or a walker (w). Stars correspond to significant shape discrimination (*p* < 0.05). Theta band in Ventral and Inferior-Frontal, as well as beta 1 in Dorso-Parietal and in Inferior-Frontal predict the shape of the visual stimuli: increasing theta synchronizations and beta1 desynchronization decrease the probability of observing a scrambled stimulus (blue area) and increase the probability of observing walker stimulus (red area). To obtain the probability of observing a kind of visual stimulus we applied the *Predict* function of the *rms* package of R. Specifically, given the coefficients estimated for the Multinomial Logistic Regression model and a vector representing putative ERSPs, the function calculated for each value of ERSP (*x* coordinate) the corresponding probability (*y* coordinate).

**Figure 6:**
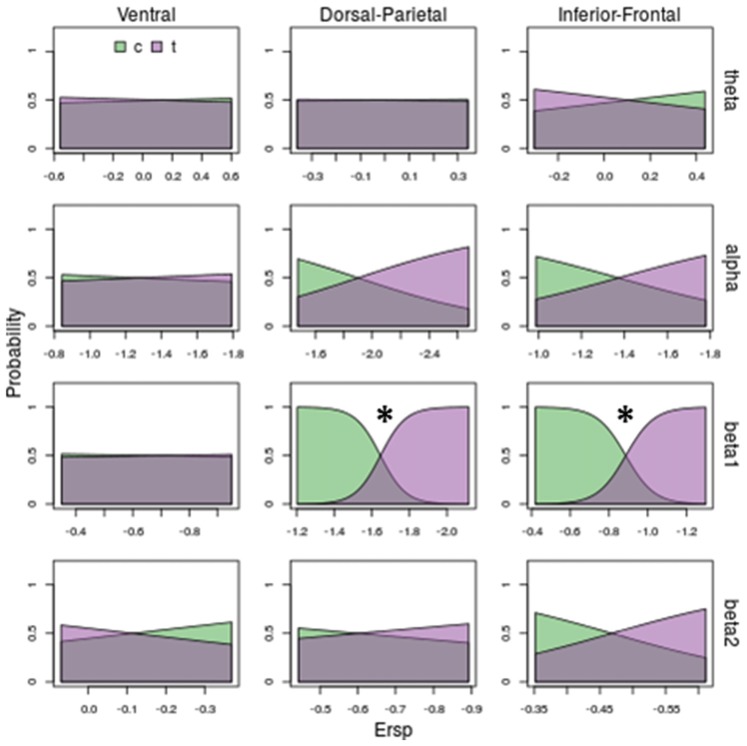
Model prediction on stimulus kinematic (centered vs. translated) based on ERSP. Rows and columns of subplots correspond respectively to different frequency bands and ROIs. In each subplot, on *x-*axis are ERSP in dB; on *y*-axis are probabilities of observing a centered (c) or a translated (t) stimulus. Stars correspond to significant models (*p* < 0.05). Beta 1 in Dorso-Parietal and in Inferior-Frontal predicts the kinematic of the stimuli: increasing beta 1 desynchronization decrease the probability of observing a centered stimulus (green area) and increase the probability of observing a translating stimulus (violet area).

**Figure 7:**
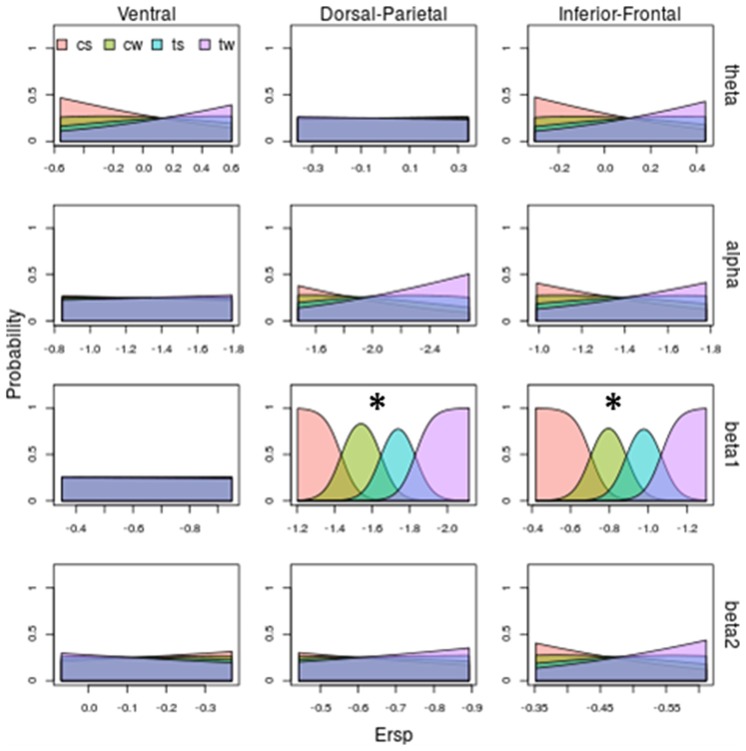
Model prediction on both stimulus shape and kinematic based on ERSP. Same legends than as Figures 5, 6. Beta1 in Dorso-Parietal and in Inferior-Frontal predicts both shape and kinematic of the visual stimulus: increasing beta 1 desynchronization predicts the observed stimulus following this order: cs (corresponding to lowest desynchronization), cw, ts and then tw (corresponding to highest desynchronization). *Abbreviations*: cs and cw for centered scrambled and walker respectively; ts and tw for translating scrambled and walker.

Figures 5–7 show separately probability to perceive a particular visual stimulus (changing in shape and/or in kinematic) as a function of ERSP pattern, for theta, alpha, beta 1 and beta 2 bands. The probability of observing a stimulus with a scrambled shape increases for low theta synchronization in Ventral and Inferior-Frontal (Figure 5, first row, red area) and progressively decreases for greater theta synchronization. Conversely, the probability of observing a coherent body structure (walker, in blue) increases with theta synchronization. Interestingly, theta better discriminates shape in Inferior-Frontal (${\text{χ}}_{\text{(1)}}^{\text{2}}$ = 45.82, *P* = 0.00000000001) than in Ventral (${\text{χ}}_{\text{(1)}}^{\text{2}}$ = 22.86, *P* = 0.00005) showing a smaller overlap between scrambled (red area) and walker (blue area). Considering now both Dorsal-Parietal (${\text{χ}}_{\text{(1)}}^{\text{2}}$ = 16.88, *P* = 0.00004) and Inferior-Frontal (${\text{χ}}_{\text{(1)}}^{\text{2}}$ = 22.66, *P* = 0.000002) ROIs (see third row of Figure 5), the probability of observing a scrambled shape is highest for low beta 1 desynchronization and progressively decreases for greater beta 1 desynchronization. Conversely, the probability to see a coherent body walker increases with beta 1 desynchronization. Instead, alpha (second row of Figure 5) and beta 2 (fourth row of Figure 5) show similar probability for scrambled and walker shapes for all ERSP values (mostly overlapped red and blue areas), therefore unable to discriminate scrambled from walker.

Concerning translation effect (see Figure 6) only beta 1 discriminates translating from centered stimuli (see third row of Figure 6): deeper beta 1 desynchronizations in Dorsal-Parietal (${\text{χ}}_{\text{(1)}}^{\text{2}}$ = 42.12, *P* = 0.00000000009) and Inferior-Frontal (${\text{χ}}_{\text{(1)}}^{\text{2}}$ = 37.14, *P* = 0.000000001) decrease the probability of a centered stimulus and increase the probability of a translating stimulus. Noticeably, translation was even better discriminated than shape, as indicated by less overlapped areas in Figure 6 compared to Figure 5.

Figure 7 shows the probability of each observed stimulus with respect to ERSP pattern and sums up all the previous observations: beta 1 is the only band discriminating both shape and translation. Specifically, as shown by third row of Figure 7, for Dorsal-Parietal (${\text{χ}}_{\text{(1)}}^{\text{2}}$ = 92.79, *P* < 0.0000000000000002) and Inferior-Frontal (${\text{χ}}_{\text{(1)}}^{\text{2}}$ = 92.19, *P* < 0.0000000000000002) ROIs, the probability of observing a centered scrambled is highest for lowest beta 1 desynchronization. Increasing beta 1 desynchronization increases the probability to see a centered walker, then a translating scrambled and finally a translating walker. Interestingly, a greater beta 1 desynchronization is necessary to predict an observed translating scrambled (blue area), while a centered walker (green area) can be predicted with a lower beta 1 desynchronization. For detailed results of the models, see Supplementary Tables S1–S3.

### Possible Effects of Ocular Movements

In this study, we controlled for possible bias due to potential eye movements induced by the translation animation. Subjects were told to anchor their gaze on the red cross; however, since half of the stimuli translated along the screen, eye movements could occur during the observation of translating stimuli, creating spurious effects on measured cortical activity. Therefore, we linearly detrended ERSP data using the mean magnitude of eye movements evaluated as the time course of the absolute value of voltages at electrode AF7 referenced to AF8 (hEye = abs(AF7 – AF8)). These electrodes have been previously considered among the most significant forehead electrodes to detect eye movements (Belkacem et al., 2014) and used to detect eye artifacts during a covert horizontal tracking task (Makin et al., 2012). Their position, near the left and right eye respectively, make their amplitudes deflect coherently with horizontal eye movements, due to the pointing direction of the corneo-retinal dipoles of the eyes, similarly to the signal provided by an horizontal electro-oculogram (Croft and Barry, 2000). Importantly, results were statistically unaffected by this correction. Moreover, especially for beta bands, we found differences between conditions with the same level of translation (i.e., cw vs. cs and tw vs. ts). Finally, when considering the mean amplitude and the 95% confident interval (CI) of ocular moments in all considered conditions (see Supplementary Figure S7 in Supplementary Material), the CI overlapped indicating the lack of significant difference between conditions.

## Discussion

The aim of the present study was to examine ERSPs during the perception of different kind of locomotor stimuli. We wanted to evaluate how the visual perception of different locomotor patterns, changing in gestalt and kinematic, modulates cortical activity, from EEG signal recorded in several areas.

More precisely, we made the specific hypothesis that forward translation affects EEG activity in the theta (4–8 Hz), alpha (8–12 Hz), beta 1 (14–20 Hz) and beta 2 (20–32 Hz) frequency bands. In support to the recorded data a multinomial logistic regressions models (MLRs) showed that the two variables tested (shape and translation) were encoded in different ways. Theta ERS increased with the more familiar shape (i.e., a walker instead of a scrambled) within Ventral and Inferior-Frontal ROIs, while translation induced significant attenuation in the power of beta (1 and 2) oscillations in Dorsal-Parietal and Inferior-Frontal ROIs. Mainly, Dorsal-Parietal and Inferior-Frontal ROIs, constituting the core of action-observation system, were the only areas capable to discriminate all the four conditions, as reflected by beta activities. The different effects of shape and kinematics variables are successively discussed in the following sections.

### Shape Effect

The result that the Inferior-Frontal theta band was particularly sensitive to body structure adds to the classical result showing a main activity located in the temporal area when manipulating shape factor. Consistent activation recurrently recorded in the superior temporal gyrus when viewing a coherent gestalt (CW) compared to scrambled animations (CS) or inverted walker (Hirai et al., 2003; Jokisch et al., 2005; Peuskens et al., 2005) supports a privileged role of the STS in body form encoding. Here, we demonstrate for the first time that theta synchronization in ventral and frontal cortices is able to discriminate a human figure from a random structure. Further, the model predicted a better discrimination of the walker from the scrambled stimuli in frontal area, an unexpected result regarding previous investigations that mainly attributed to STS a role in shape recognition (Hirai et al., 2003; Jokisch et al., 2005; Peuskens et al., 2005). This outcome supports the idea of a significant role of motor regions in the specific theta band for body figure encoding.

Because centered scrambled stimulus does not contain gestalt-like patterns, one may have predicted the lack of sensorimotor activity for scrambled display. Instead, the desynchronization of beta band, even if reduced, was still present for scrambled stimuli. Nevertheless, a CS stimulus still displays PLD motion compatible with human kinematic, especially the dot located on the lower limbs. Precisely, the centered scrambled preserved local motions compatible with motor representation, namely the 2/3 power law (Lacquaniti et al., 1983), also present during treadmill locomotion (Ivanenko et al., 2002). For instance a cloud of dots moving along elliptical trajectories strictly according to this motor rule is enough to activate dorsal premotor and supplementary motor areas (Dayan et al., 2007; Meirovitch et al., 2015). Similarly, the present local oscillations produce by central pattern generator can generate ERD modulation during motion observation. A recent study performed with patient with lesion to the form visual pathways also agrees with the idea that form cues are not critical for biological motion perception (Gilaie-Dotan et al., 2015) and that observer can still discriminate locomotion direction or identify living being from spatially scrambled displays that contain solely local biological motion cues (Sumi, 1984; Pavlova, 1989; Chang and Troje, 2008). Nonetheless, beta 1 band suppression in fronto-parietal areas was stronger when the stimulus displayed a coherent body structure compared to the scrambled version suggesting that human body geometry improves the sensorimotor integration of the visual input.

### Translation Effect

Surprisingly we did not find any translation effect on alpha oscillations, a frequency band classically considered as a correlate of the Action-Perception network activity. Indeed, Mu suppression refers to an attenuation in the alpha frequency range (8–13 Hz) recorded over sensorimotor cortex both during action execution and action observation (Cochin et al., 1999; Babiloni et al., 2002; Pineda, 2005; Hari, 2006; Orgs et al., 2008; Perry and Bentin, 2009). A recent investigation by Kraskov et al. (2014) recorded mirror neurons in area F5 of macaque monkeys while they observed a reach-to grasp action may however explain such inconsistency. These authors found that the local field potential activity in F5 neurons recorded in the beta-frequency range (15–23 Hz) was attenuated during action observation. More precisely the power in the 15–23 Hz beta range recorded in area F5 was significantly attenuated in the first 300 ms after movement onset. Moreover, Urgen et al. (2013) found alpha band suppression for both human and robot action observation. All together, these experimental evidences indicate a lack of sensitivity of alpha band for human action perception, at least for early sensory stages of action visual processing. In support to this possibility, a recent investigation performed during observation of human gait suggested that early alpha ERS contributes to a general clearance of noise or distracting event in order to selectively update relevant incoming information and increase involvement of cognitive resources (Zarka et al., 2014).

Comparison between centered and translated displays showed greater beta 1 suppression in the dorso-parietal and inferior-frontal ROIs for translating compared to centered stimuli. This confirms that sensorimotor areas are precociously involved in differentiating between movement kinematics (Press et al., 2011; Di Dio et al., 2013; Meirovitch et al., 2015). This also suggests that the translating scrambled (a sort of “blob in motion” as reported a posteriori by the participants) brings significant sensorimotor information in the motor resonance process, probably because local intrinsic movements are interpreted as the cause of extrinsic translating motion (see Thurman and Lu, 2013).

We found that beta band suppression was more pronounced toward medial and posterior locations (centro-parietal locations) than in central or fronto-central electrodes. Previous investigation showed sensorimotor suppression during movement preparation and execution (Pfurtscheller et al., 1997), motor imagery (Pfurtscheller et al., 2006) or action observation (Muthukumaraswamy and Johnson, 2004; Ulloa and Pineda, 2007) in a somatotopic way. For instance hand movement is accompanied by a central-lateral alpha rhythm suppression, whereas feet movements by centro-medial alpha rhythm suppression (Pfurtscheller et al., 1997). The present stronger beta rhythms suppression along medial locations, i.e., those most likely overlaying somatosensory areas of the feet and legs (Penfield and Rasmussen, 1950) corroborates the link between perception and action systems.

It has been recurrently proposed that sensorimotor ERD reflects the transformation of perceptual representations to executable actions (Pineda, 2005). The finding that beta ERD responses in expert dancers viewing dance movements are stronger than in non dancers observing similar movements (Orgs et al., 2008) also agrees with the idea of a role of the motor system in visual perception of action. According to this, the present significant effect of translation on the recorded sensorimotor ERD indicates that body translation, in contrast to treadmill walking, facilitates the matching between visual input and motor representation. Accordingly, when walking on the spot optical flow is missing. Moreover, locomotor reactions in response to the passive displacement of the base of support is not compatible with integration of the relative motion of the head, the torso and the eyes, that is crucial to build sensorimotor (Berthoz et al., 1992; Grasso et al., 1998; Pham et al., 2011) and navigational components of voluntary locomotion (Plank et al., 2010; Gramann et al., 2011; Chiu et al., 2012). The artificial sensory context created by the treadmill locomotion could degrade the matching between visual input incongruent with stored representation.

Further, even if the observation of a walker without definite goal (as walking on the spot) might activate the motor cortex (Saunier et al., 2013), the coupling between perception and action system is enhanced when a goal is present (Umiltà et al., 2001; Rizzolatti and Craighero, 2004). However, inference on walker’s goal is easier if the observer can make sensorimotor predictions about the current state of the actor-environment system on the basis of previous sensory input generated during natural body translation. A simple illustration of this is the difficulty one has to perform covert artificial tasks, as locomotion on a treadmill compared to real forward locomotion, or when sensory feedback information is lacking (see Courtine and Pozzo, 2004). Even if locomotion on the spot may elicit a vivid impression of translational motion (Pavlova et al., 2002; Saygin et al., 2010; Viviani et al., 2011), the translating visual stimuli would assign more easily a spatial goal to the perceived motion and thus would facilitate the recall of specific kinematic details. Accordingly, Thurman and Lu (2013) found that introducing extrinsic translational motion congruent with the direction implied by the intrinsic movements increased the perceived animacy of spatially scrambled walkers. These authors proposed that extrinsic motion would convey a clearer impression of directionality and intentional behavior in the moving agents.

At last, because cyclical locomotor behavior of most vertebrates relies on similar neural networks (Grillner and Zangger, 1979; Lacquaniti et al., 1999; Dominici et al., 2011) and because successful social interactions and survival of species rely on efficient visual processing of biological motion, one may predict that forward displacement will represent a prior knowledge and “a strong attractor” for both human and animal visual system. A recent behavioral study showing human newborns preference for the translated locomotion supports the existence of a privileged neural imprint constraining the visual perception for horizontal displacements (Bidet-Ildei et al., 2014).

One possible hypothesis to explain the present impact of the translational component on sensorimotor ERD would be that artificial “on the spot treadmill locomotion” would mainly rely on visual decoding mechanism. The occipital cortex and the STS would ensure the visual recognition of cyclical displacements of each body part controlled by central pattern generators similar in several species (Orlovskiĭ et al., 1999) whereas the horizontal displacement would be necessary to motor resonance, that a congruent body structure associated to biological kinematic would improve.

## Conclusion

Our results suggest that body translation prevails compare to pictorial information in the process mapping the visual input onto stored representations of movements. Nevertheless, the exact manner by which perception of locomotion matches the motor system remains elusive and requires further neurophysiological investigations. Indeed, one central question is how motor resonance initially identified for hand movement is activated for cyclical lower limb movement mainly encoded in the spinal cord.

## Author Contributions

TP and AK conceived and designed the experiments. AK performed the experiments. CC, AI and SP analyzed the data. TP, CC, AI, GS and SP wrote the article. TP supervised the whole project.

## Conflict of Interest Statement

The authors declare that the research was conducted in the absence of any commercial or financial relationships that could be construed as a potential conflict of interest.
