# Supplementary material for: Natural Translating Locomotion Modulates Cortical Activity at Action Observation
Source: Front Syst Neurosci. 2017 Nov 7;11:83. doi: 10.3389/fnsys.2017.00083 (PMC5681993; doi:10.3389/fnsys.2017.00083)
Supplement: Supplementary file 1 [file Data_Sheet_1.pdf]

Supplementary Material

Natural Translating locomotion modulates cortical activity at action observation

T. Pozzo[1-3*], A Inuggi[2], A Keuroghlanian[2], S Panzeri[4], G Saunier[5], C Campus[6]

[1] Istituto Italiano di Tecnologia, Centro di Neurofisiologia Traslazionale, Ferrara, Italy
[2] Unit of Robotics, Brain and Cognitive Sciences, Istituto Italiano di Tecnologia, Center for Human Technologies, Genova, Italy
[3] INSERM-U1093, CAPS, Campus Universitaire, Dijon, France
[4] Laboratory of Neural Computation, Center for Neuroscience and Cognitive Systems @UniTn, Istituto Italiano di Tecnologia, 38068 Rovereto, Italy
[5] Laboratorio de Cognição Motora, Departamento de Anatomia, Universidade Federal do Pará, Belém, Brasil
[6] U-VIP Unit for Visually Impaired People, Istituto Italiano di Tecnologia, Tecnologia, Center for Human Technologies, Genova, Italy

*** Correspondence:**

Fondazione Istituto Italiano di Tecnologia Centro di Neurofisiologia traslazionale c/o sezione, Fisiologia Umana Via Fossato di Mortara, 17-19 44121 Ferrara, Italy

1 Supplementary Methods

1.1 Details about statistical analyses

1.1.1 Details about comparing ERSP in specific frequency bands

We considered a time window between 80 and 800 ms to exclude possibly spurious findings due to very-early and very-late effects.

Then, for each subject, ROI and frequency band, we considered a time window from 100 ms before to 100 ms after the latency (time) of the extreme value.

For each subject, band and condition we used the average ERSP of this 200 ms time window realigned to the extreme for the statistical comparisons and for the development of the predictive models.

1.1.2 Details about the predictive models

1.1.2.1 Fitting the models

First, we fitted the models to our data. As a model family, we preferred multinomial logistic regressions (MLRs), which are more generalizable and robust, with performance better or comparable, compared with linear classification models (Press and Wilson, 1978).

MLR is a classification method that generalizes logistic regression to multiclass problems, i.e. with more than two possible discrete outcomes.

MLR predicts the probabilities of the different possible outcomes of a categorically distributed dependent variable (such as visual stimuli), given a set of independent variables (such as ERSP in frequency bands).

1.1.2.2 Evaluating the predictive ability of the models

As a measure of the performance of the models we used Somers' D (Somers, 1962) which is a rank correlation between predicted probabilities and observed responses. When D = 0 the model is making random predictions; when D = 1 the predictions are perfectly discriminating.

1.1.2.3 Validating the models

1.1.2.3.1 The problem of overestimating the predictive ability of the model (over-fitting)

The validation procedure is a crucial step to ensure that the results and the performance of the fitted models can be generalized to new datasets. In fact, fit measures are usually biased by over-fitting , i.e. they indicate better fit, or less prediction error than is really the case. Prediction error refers to the discrepancy or difference between a predicted value (based on a model) and the actual value. In the standard regression situation, prediction error refers to how well our regression equation predicts the outcome variable scores of new cases based on applying the model (coefficients) to the new cases' predictor variable scores. When dealing with a single sample, typically the residuals are a reflection of this prediction error. However, because of over-fitting, these errors are biased downward (less prediction error) due to the actual outcome variable values being used to create the regression equation (i.e. the prediction model).

1.1.2.3.2 Obtaining unbiased estimation of the predictive ability of a model

To obtain an unbiased estimation of model performance, the experimenter can apply the fitted model to a new and independent dataset, observing how a measure of performance changes. Internal validation (Steyerberg et al., 2001) is an alternative way to address this over-fitting bias and is based on split-sample, cross-validation and bootstrapping methods involving only the original dataset; therefore it doesn't require new experiments. Among different internal validation strategies, we chose bootstrap, which was reported to have the best performances (Steyerberg et al., 2001).

1.1.2.3.3 The bootstrap strategy

The bootstrap strategy verifies how a performance measure changes by applying the fitted model to new datasets built by random sampling with replacement the original dataset: **if the prediction**

measure does not change much, it is assumed that the performance of the model is reproducible for other data.

1.1.2.3.4 Removing the Harrel's Optimism to get unbiased estimation

To test how much the Somers' D of the models changes for new datasets we used the Harrell's "Optimism" measure O_H (Harrell, 2015). For each multinomial regression, we initially fitted the regression model M using all of the original data and we calculated the corresponding Somers' D.

In the next step, for each repetition b we generated a bootstrap sample by drawing at random and with replacement from the original data, we fitted to the bootstrap sample a model M* with new coefficients and we calculated D_{boot} (b), i.e. the corresponding Somers' D. Conversely, we calculated D_{orig} (b), i.e. the Somers' D obtained by applying M* to the original data. For each repetition b, the Harrell's "Optimism" measure O_H (b) is defined to be D_{boot} (b) - D_{orig} (b). An "Optimism" smaller than 0.05 indicates that the original model M is consistent in its performance: in this case there is no degradation in predictive power when the original model is applied to the bootstrap data set, i.e. the performance of M* on the original data is comparable to that of M. This procedure was repeated B=10000 times and the average O_H was calculated over all repetitions:

$$O_H = \frac{\sum_{b=1}^{B} D_{boot}(b) - D_{orig}(b)}{B}$$

The corrected Somers' D, i.e., $D_{corr} = D_{app} - O_H$ gives a robust estimate of the internal validity of the model, with O_H acting as a penalty term for over-fitting. Both the fitting and the validation of the models were implemented by the rms package (Harrell, 2015) of the R environment (R Core Team, 2017).

2 Supplementary Figures

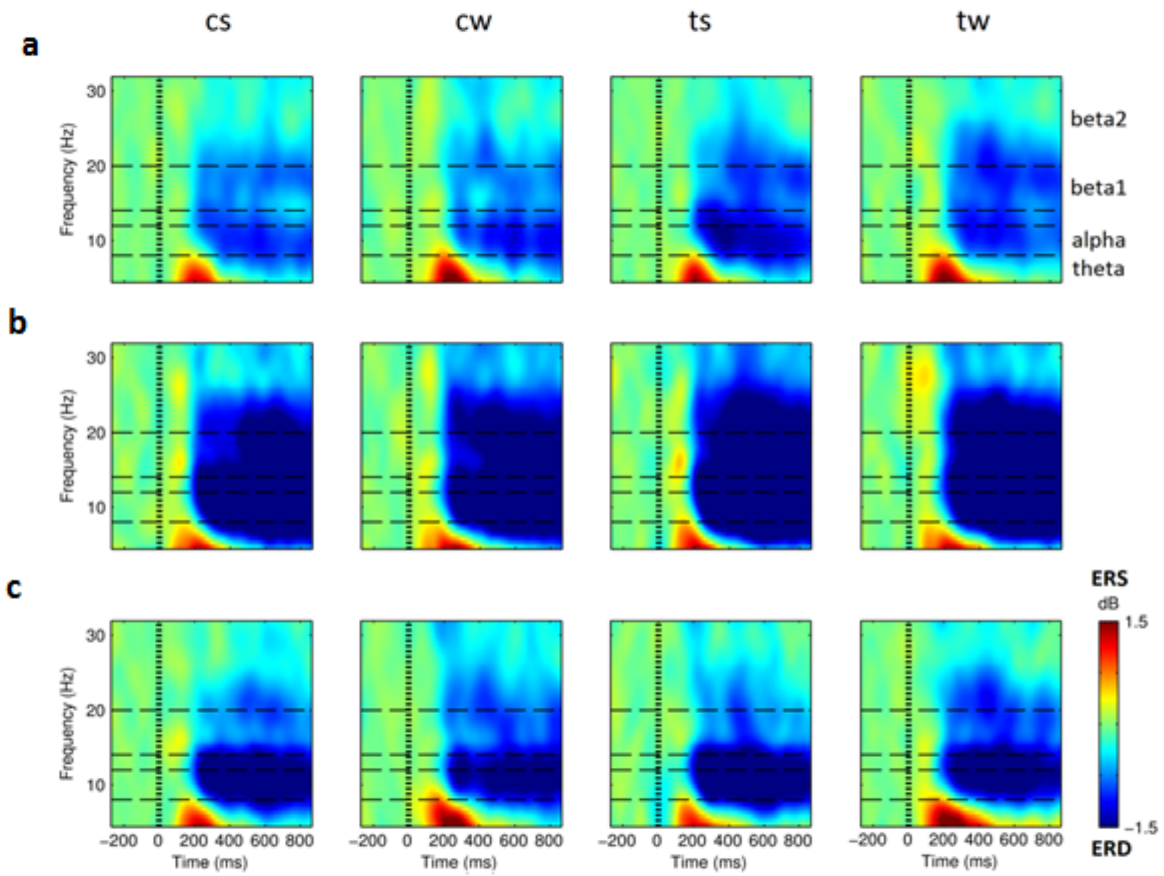

Figure S 1. ***Spectrograms obtained from different ROIs and conditions.*** Event Related Spectral Perturbations (ERSP) are expressed at different times t (x axis) and frequencies (y axis) as in dB, i.e. Log(Power(t)/Power during baseline). Blue indicates event related desynchronizations (ERD), i.e. power decreases; green indicates null variations, while red indicates event related synchronizations (ERS), i.e. power increases. In all the ROIs and conditions we found pronounced initial ERSs, mainly involving low frequencies, followed by extended ERDs mainly involving high frequencies. (a) ERSP in Ventral. (b) ERSP in Dorsal-Parietal. (c) ERSP in Inferior-Frontal.

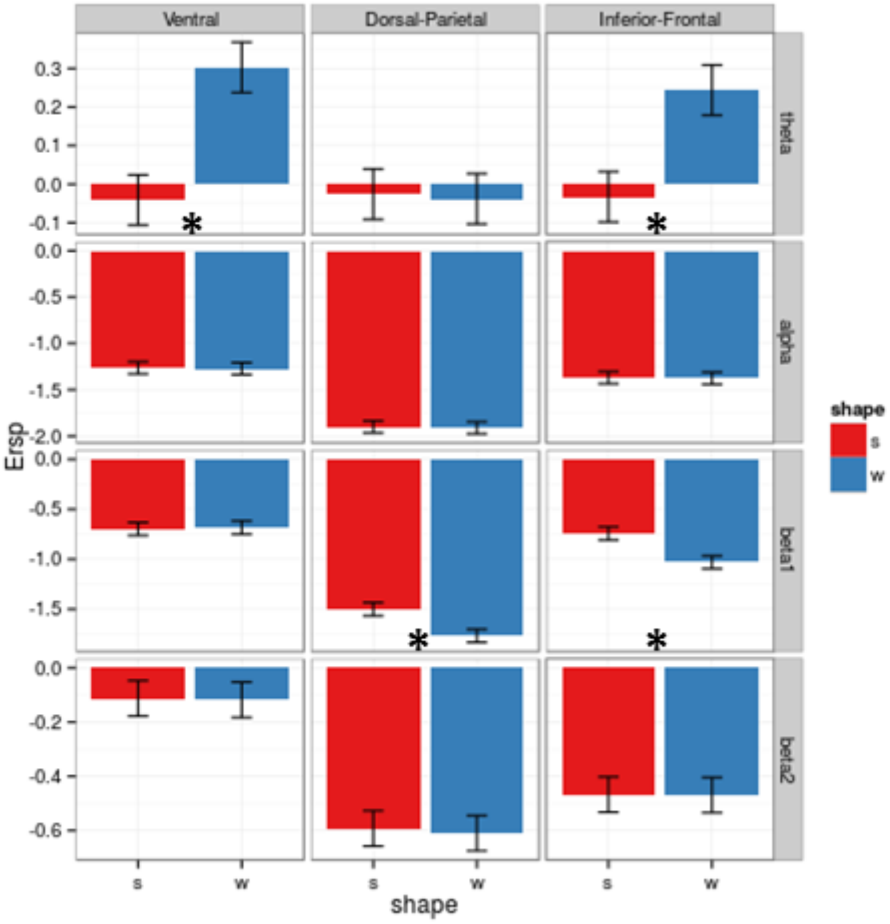

Figure S 2. ***Results of post-hoc ANOVAs considering Ersp: shape effect.*** Rows of subplots correspond to different bands, columns correspond to regions of interest. On x axis are levels of shape factor (scrambled, walker), on y axis are event related spectral perturbations (Ersp) in dB. Bars correspond to means and standard errors. Stars correspond to significant differences ($p<0.05$). Shape affects theta in Ventral and Inferior-Frontal, as well as beta1 in Dorsal-Parietal and Inferior-Frontal: a walker shape corresponded to higher theta synchronizations and higher beta1 desynchronization with respect to a scrambled shape.

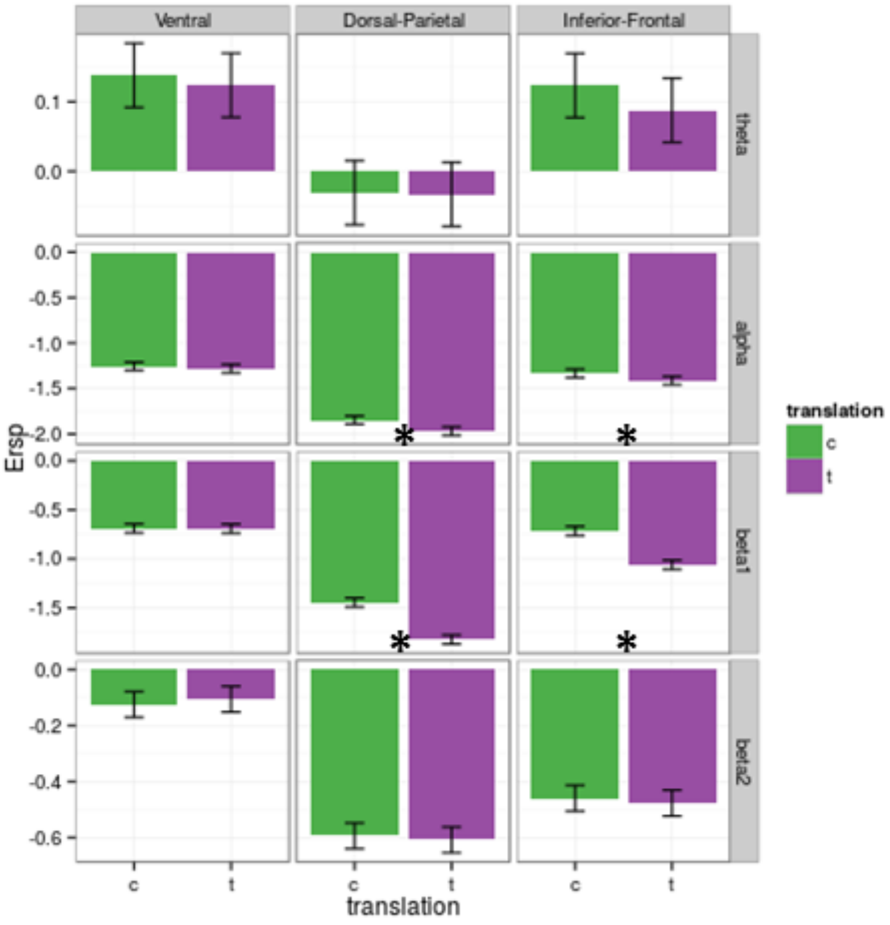

Figure S 3. ***Results of post-hoc ANOVAs considering Ersp: shape effect.*** Rows of subplots correspond to different bands, columns correspond to regions of interest. On x axis are levels of shape factor (scrambled, walker), on y axis are event related spectral perturbations (Ersp) in dB. Bars correspond to means and standard errors. Stars correspond to significant differences ($p<0.05$). Shape affects theta in Ventral and Inferior-Frontal, as well as beta1 in Dorsal-Parietal and Inferior-Frontal: a walker shape corresponded to higher theta synchronizations and higher beta1 desynchronization with respect to a scrambled shape.

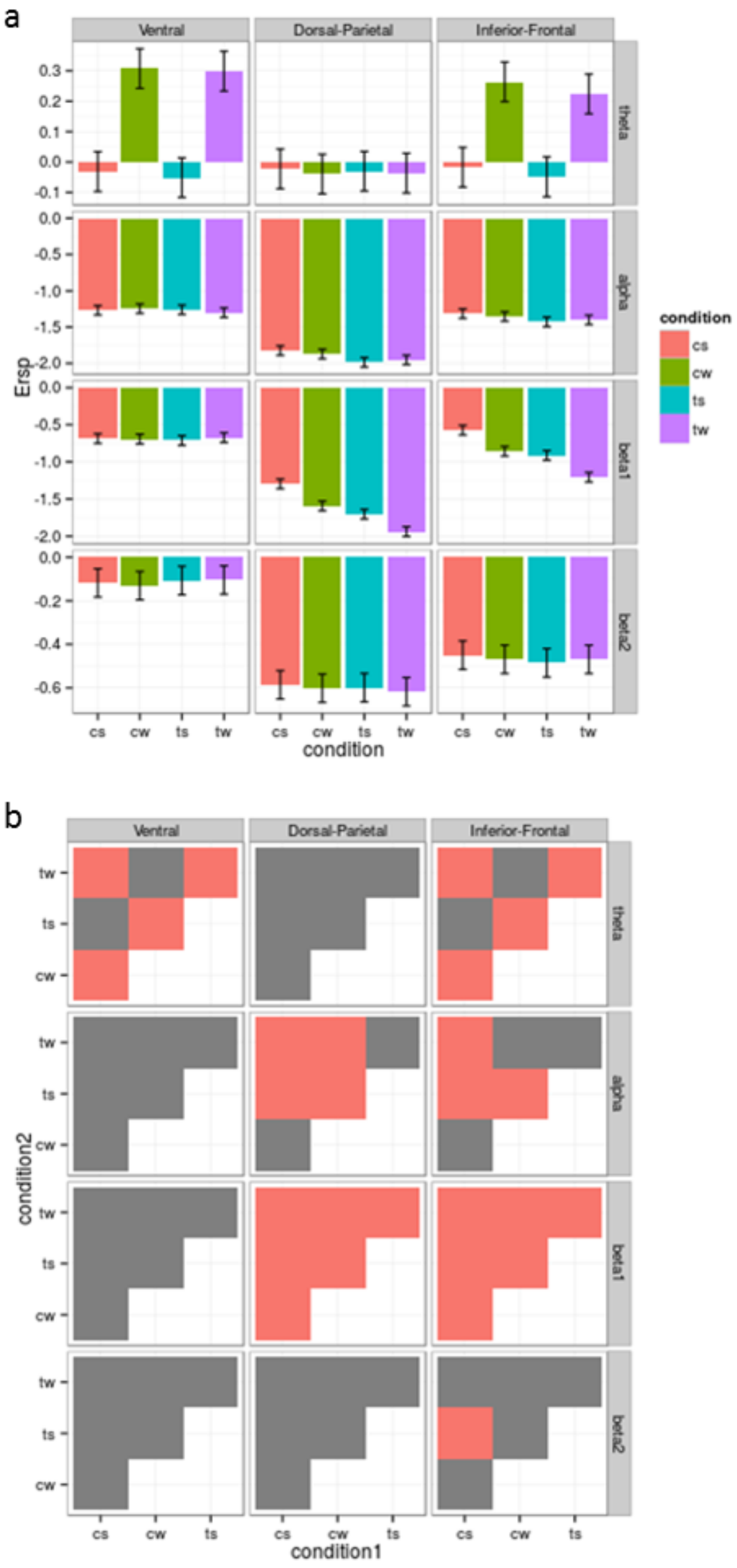

Figure S 4. ***Interaction between shape and condition effects.*** (a) Rows of subplots correspond to different bands, columns correspond to regions of interest. On x axis are conditions (centered scrambled, centered walker, translating scrambled, translating walker), on y axis are event related

spectral perturbations (Ersp) in dB. Bars correspond to means and standard errors. (b) Results of post-hoc ANOVAs: comparing conditions. Rows of subplots correspond to different bands, columns correspond to regions of interest. Each subplot represent pairwise compairsons beween Ersp in different conditions (centered scrambled, centered walker, translating scrambled, translating walker) Red squares correspond to significant differences ($p<0.05$). Theta in Ventral and in Inferior-Frontal showed higher synchronizations for stimuli with a walker shape (cw, tw) compared with scrambled shape (cs, ts). Alpha in Dorsal-Parietal and in Inferior-Frontal showed higher desynchonizations for translating stimuli (ts, tw) compared with centered stimuli (cs, cw). Beta 1 showed differences among all considered conditions. Beta2 showed only higher desynchronization for the translating scrambled than for the centered scrambled stimulus.

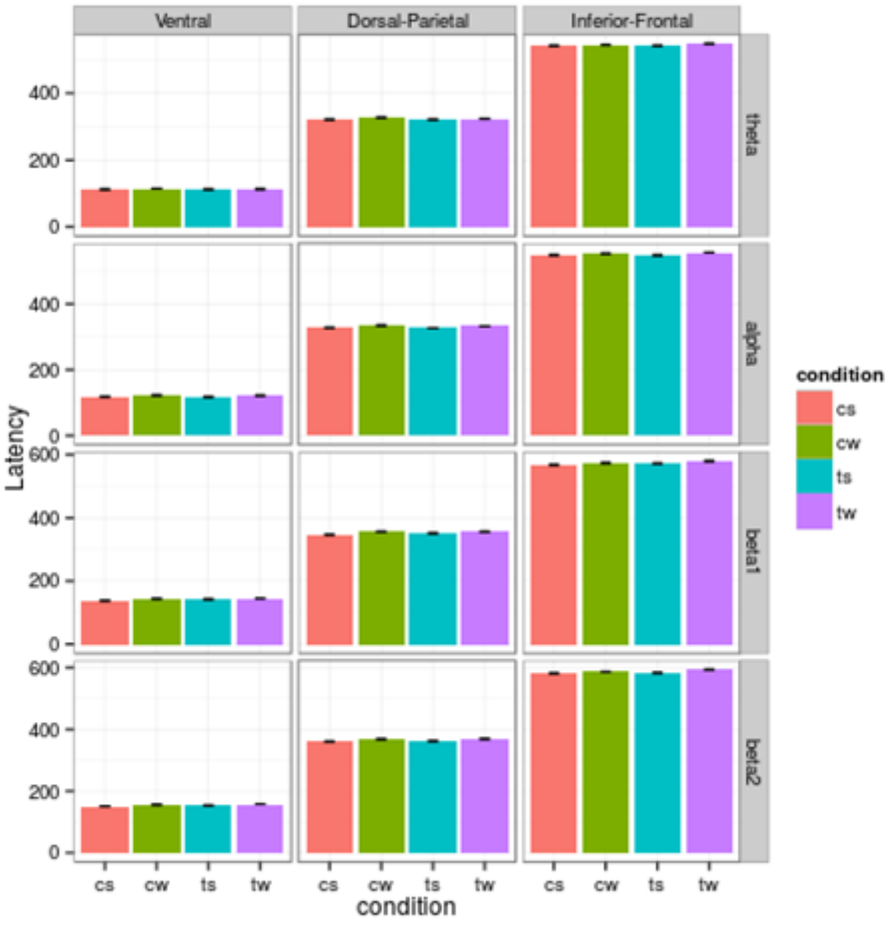

Figure S 5. ***Latencies corresponding to Ersp reaching extreme values: comparing conditions.*** Rows of subplots correspond to different bands, columns correspond to regions of interest. On x

axis are conditions (centered scrambled, centered walker, translating scrambled, translating walker), on y axis are latencies in ms. Bars correspond to means and standard errors.

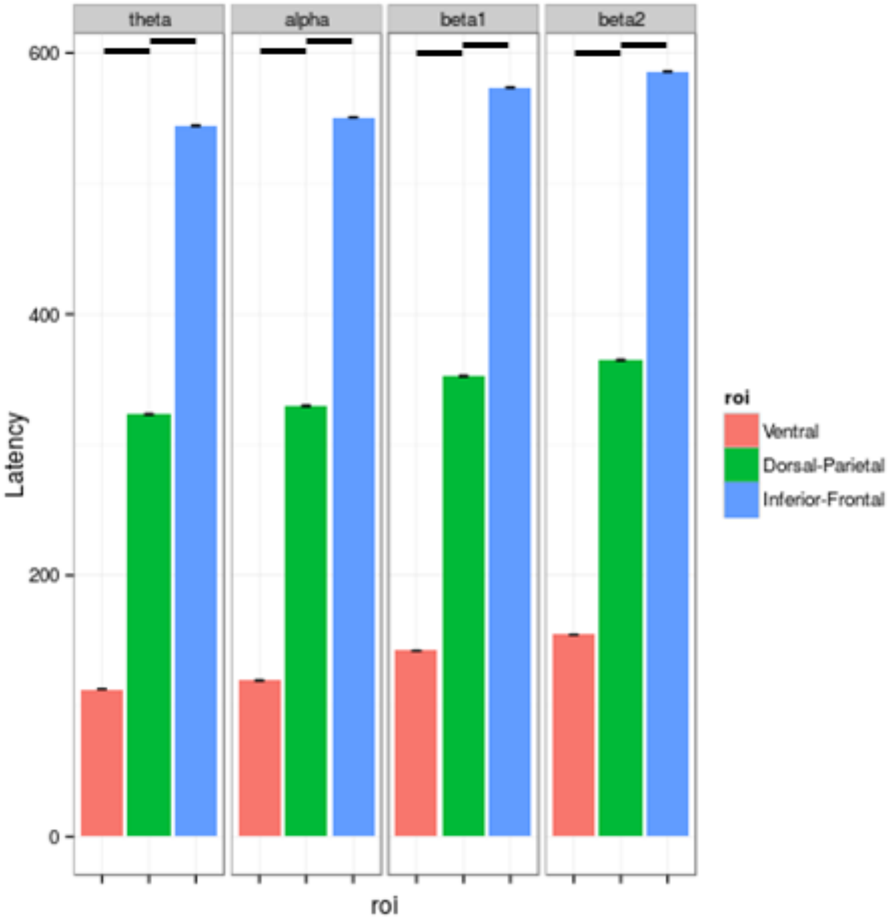

Figure S 6. ***Results of post-hoc ANOVAs considering latencies: the effect of regon of interest.*** Subplots correpond t different bands. On x axis are rois, on y axis are latencies in ms. Bars correspond to means and standard errors. Stars correspond to significant differences ($p<0.05$). Similarly for all bands, latencies increase following the order Ventral, Dorsal- Prietal, Inferior-Frontal.

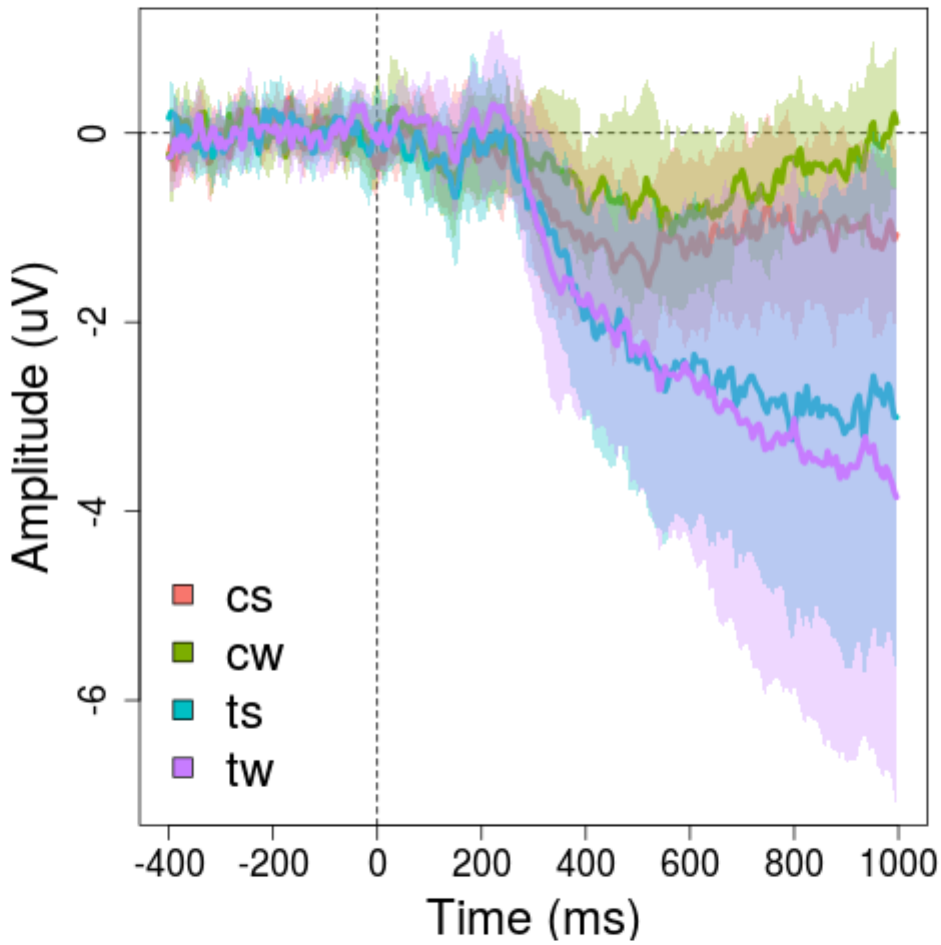

Figure S 7. ***Interaction between shape and translation effects cannot be merely explained by ocular movements.*** For each condition, mean amplitude of ocular movements is represented by colored line, while 95% CI is represented by the area covered by transparent version of the same color. The conditions in which the stimulus was translating presented higher amplitude of ocular movements, but with a huge variability among subjects, reflected by CIs strongly overlapped between conditions.

3 Supplementary Tables

Table S 1. ***Global statistics of the models.*** For each model, we report the model likelihood ratio chi-square (with the corresponding degree of freedom), the P-value, the Somers' D (i.e. a measure of discriminating performance) of the model fitted on original data, the Harrell's Optimism estimated through bootstrap technique and the unbiased Somers' D corrected by subtracting the Optimism form the initial D. All models showed a high discriminating performance (D), even after the subtraction of Harrell's Optimism.

Predicted feature	band	ROI	Chisq(1)	P	D0	O	Dcorr
shape	theta	Ventral	20.86	<0.0001	0.80	0.05	0.75
		Inferior-Frontal	45.82	<0.0001	0.91	0.01	0.90
	beta 1	Dorsal-Parietal	16.88	<0.0001	0.80	0.03	0.77
		Inferior-Frontal	22.66	<0.0001	0.82	0.01	0.81
translation	beta 1	Dorsal-Parietal	42.12	<0.0001	0.95	0.08	0.87
		Inferior-Frontal	37.14	<0.0001	0.93	0.07	0.86
embodiment	beta 1	Dorsal-Parietal	92.79	<0.0001	0.96	0.07	0.89
		Inferior-Frontal	92.19	<0.0001	0.89	0.07	0.82

Table S 2. ***Coefficients of the models.***

Predicted feature	band	ROI	Prediction	Coef	S.E.	Wald Z	P
shape	theta	Ventral	w vs s	5.73	1.63	3.49	0.0005
		Inferior-Frontal	w vs s	25.83	8.41	3.07	0.003
	beta 1	Dorsal-Parietal	w vs s	-5.58	1.64	-3.4	0.001
		Inferior-Frontal	w vs s	-7.47	2.11	-3.55	0.0004
translation	beta 1	Dorsal-Parietal	t vs c	-14.93	4.46	-3.35	0.001
		Inferior-Frontal	t vs c	-13.63	4.23	-3.22	0.002
embodiment	beta 1	Dorsal-Parietal	cw vs cs	-32.36	6.44	-5.03	< 0.0001
			ts vs cw	-37.12	7.26	-5.11	< 0.0001
			tw vs ts	-41.24	8.02	-5.14	< 0.0001
		Inferior-Frontal	cw vs cs	-15.98	3.24	-4.94	< 0.0001
			ts vs cw	-20.17	3.95	-5.11	< 0.0001
			tw vs ts	-24.26	4.67	-5.19	< 0.0001

Table S 3. ***Results of ANOVAs on latencies of ERSP extreme values.*** Degrees of freedom are in parentheses. Significant effects (P<0.05) are in bold.

Effect	Band	F	p	ges
roi (2,24)	theta	355.58	0.000000000000000001	0.84
	alpha	85.01	0.00000000001	0.59
	beta1	672.29	0.0000000000000000000008	0.86
	beta2	65.50	0.0000000001	0.42
shape (1,12)	theta	0.06	0.81	0.00056
	alpha	0.03	0.86	0.00037
	beta1	0.17	0.69	0.0018
	beta2	0.03	0.87	0.00026
translation (1,12)	theta	0.00	0.98	0.0000046
	alpha	0.00	0.99	0.0000013
	beta1	0.04	0.84	0.00049
	beta2	0.00	0.95	0.000042
roi*shape (2,24)	theta	0.01	0.99	0.000058
	alpha	0.00	1	0.000016
	beta1	0.01	0.99	0.000068
	beta2	0.00	1	0.0000082
roi*translation (2,24)	theta	0.01	0.99	0.000072
	alpha	0.01	0.99	0.000020
	beta1	0.01	0.99	0.000084
	beta2	0.00	1	0.00001
shape*translation (1,12)	theta	0.00	0.97	0.0000046
	alpha	0.00	0.98	0.0000051
	beta1	0.01	0.92	0.000034
	beta2	0.00	0.98	0.0000026
roi*shape*translation (2,24)	theta	0.01	0.98	0.000072
	alpha	0.01	0.99	0.000020
	beta1	0.01	0.99	0.000084
	beta2	0.00	1	0.00001
